# Single-cell RNA-sequencing of the brain

**DOI:** 10.1186/s40169-017-0150-9

**Published:** 2017-06-08

**Authors:** Raquel Cuevas-Diaz Duran, Haichao Wei, Jia Qian Wu

**Affiliations:** 1 The Vivian L. Smith Department of Neurosurgery, McGovern Medical School, The University of Texas Health Science Center at Houston, Houston, TX 77030 USA; 2 Center for Stem Cell and Regenerative Medicine, UT Brown Foundation Institute of Molecular Medicine, Houston, TX 77030 USA

**Keywords:** Single-cell RNA-sequencing, Brain, Heterogeneity, Bioinformatic analyses

## Abstract

Single-cell RNA-sequencing (scRNA-seq) is revolutionizing our understanding of the genomic, transcriptomic and epigenomic landscapes of cells within organs. The mammalian brain is composed of a complex network of millions to billions of diverse cells with either highly specialized functions or support functions. With scRNA-seq it is possible to comprehensively dissect the cellular heterogeneity of brain cells, and elucidate their specific functions and state. In this review, we describe the current experimental methods used for scRNA-seq. We also review bioinformatic tools and algorithms for data analyses and discuss critical challenges. Additionally, we summarized recent mouse brain scRNA-seq studies and systematically compared their main experimental approaches, computational tools implemented, and important findings. scRNA-seq has allowed researchers to identify diverse cell subpopulations within many brain regions, pinpointing gene signatures and novel cell markers, as well as addressing functional differences. Due to the complexity of the brain, a great deal of work remains to be accomplished. Defining specific brain cell types and functions is critical for understanding brain function as a whole in development, health, and diseases.

## Introduction

Single-cells are the fundamental units of unicellular and multicellular organisms. Every single-cell in an organism is unique in its transcriptome, epigenome, and its local microenvironment. Even genetically identical cells display stochastic gene expression due to random fluctuations in the mechanisms driving and regulating transcription and translation [1, 2]. The underlying heterogeneity within cells is a fundamental property of cellular systems for homeostasis and development [3]. Different cell types specialize in the execution of specific tasks [4].

Next-generation sequencing technologies, such as RNA-sequencing, have become a standard for querying gene expression [5, 6]. However, gene expression levels obtained through such ensemble-based approaches yield expression values averaged across large populations of input cells, masking cellular heterogeneity. Recent experimental advances have allowed the isolation of single-cells and the generation of cDNA libraries from low amounts of RNA. Through scRNA-seq researchers are able to determine expression profiles in single-cell resolution. Since the introduction of scRNA-seq [7], the number of single-cell experiments has greatly increased. scRNA-seq has demonstrated to be a powerful tool to identify and classify cell subpopulations [8], characterize rare or small subpopulations [9], and trace cells along dynamic cellular stages, such as during differentiation [10].

The mammalian brain is a complex tissue that contains a large number of specialized cells with differences in morphology, connectivity, and functions [11–13]. Brain cells have been classified by location, morphology, electrophysiological characteristics, target specificity, molecular markers and gene expression patterns [14–17]. Single-cell analysis is critical for studying the brain since small differences in a seemingly homogeneous population may explain issues relating cells to learning, memory, and other cognitive functions [18]. scRNA-seq makes it possible to understand the heterogeneity and the regulatory networks within brain cells at the transcriptome level.

The general framework of a scRNA-seq experiment consists of: single-cell isolation, cell lysis, mRNA capturing, mRNA reverse transcription into cDNA, cDNA amplification, library preparation, and sequencing [19]. Herein, we will review recent research in brain cells with scRNA-seq. In the first two sections, we will discuss the advances and limitations of the methods for single-cell isolation and library generation. Section three will summarize the analysis methods of scRNA-seq data. Subsequently, we will discuss recent and relevant findings derived from scRNA-seq of brain cells. Finally, we will highlight future applications and challenges of scRNA-seq in brain.

## Single-cell isolation protocols

The first important step in scRNA-seq is to isolate single-cells from tissues keeping their expression patterns as accurate as possible. Several technologies have been used, such as: FACS (Fluorescence-activated cell sorting), MACS (Magnetic-activated cell sorting), LCM (Laser capture microdissection), manual cell picking and microfluidics. Depending on the nature of samples, different methods may be more suitable for single-cell isolation in distinct samples. In this section, we will discuss some methods used for isolating brain cells.

**Fig. 1:**
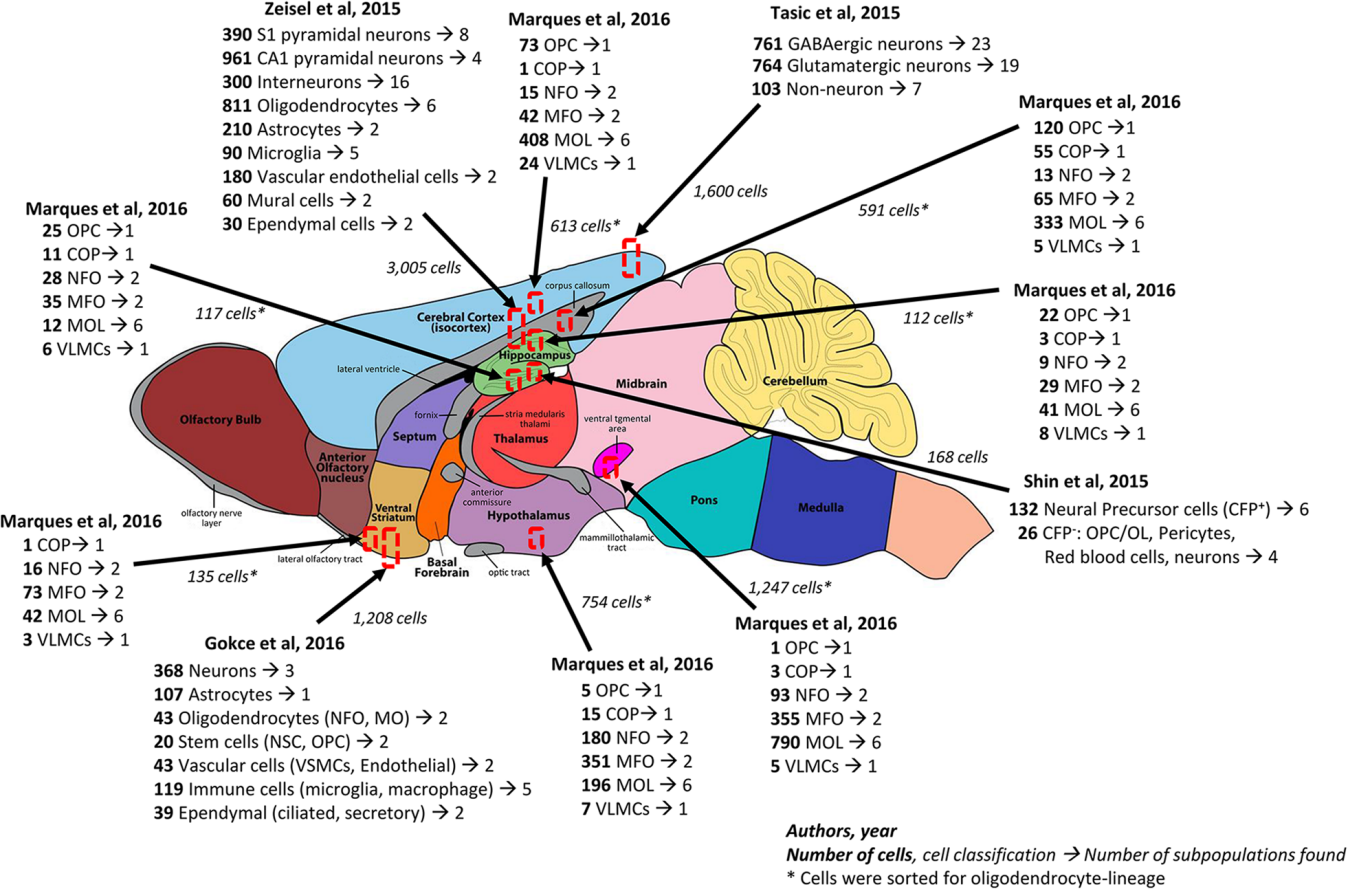
Selected relevant scRNA-seq studies revealing brain heterogeneity. Recent high throughput brain scRNA-seq studies indicate that mouse brain is composed of a large diversity of specialized cell subpopulations. *Arrows* indicate the sample collection region and the number of isolated cells. The *numbers to the left* represent the quantity of cells belonging to each global cell type. The *numbers to the right* represent the quantity of subpopulations found within each global cell type. *Asterisks* indicate cells were enriched for oligodendrocyte-lineage. Brain model schematic obtained from GENSAT (Gene Expression Nervous System Atlas) [120, 125]

Fluorescence-activated cell sorting and MACS are widely used methods to isolate single-cells. FACS can purify single-cells based on cell size, granularity and fluorescence. Surface markers are different in individual cells, so FACS can isolate specific cells stained with different fluorescently-tagged monoclonal antibodies [20]. In brain cell research, cells have been labelled with different markers. For example, Tasic et al. [21] used combinations of *Snap25*, *Slc17a7*, and *Gad1* to find subpopulations in the primary visual cortex as listed in Table 1 and depicted in Fig. 1. Similarly, Llorens-Bobadilla et al. [22] labelled cells with *GLAST*/*Prom1* and *PSA*-*NCAM* to dissect populations in the subventricular zone. Although FACS is a highly efficient method to isolate single-cells, it has its limitations: not all cell types have their own specific gene markers [23], and the binding of fluorescently-tagged monoclonal antibodies to cells might alter their function [24]. One major disadvantage of FACS is its low cell throughput rate. Even high-speed sorters will yield a few thousand cells per second [25]. Since many experiments require large number of cells, sorting runs may take long times posing quality issues to sorted cells. MACS is another method used to isolate single-cells [26]. The cells are isolated by biodegradable iro based nanobeads bound with specific cell surface antibodies. Although MACS can produce high yield single-cells and is widely used, one of its main limitations is that antibody-coated magnetic beads are specific only for cell surface markers.

**Table 1:** Overview of recent brain scRNA-seq studies

Brain region	Isolation method	RT and cDNA amplification	# of cells sequenced	Major populations identified	# of cells in population	Subpopulations identified	Refs.
Primary visual cortex	FACS	SMARTer	1600	GABAergic neurons	761	23	[21]
				Glutamatergic neurons	764	19	
				Non-neuronal	103	7	
Somatosensory cortex, hippocampal CA1	Fluidigm, FACS	STRT/C1	3005	S1 pyramidal neurons	390	8	[8]
				CA1 pyramidal neurons	961	4	
				Interneurons	300	16	
				Oligodendrocytes	811	6	
				Astrocytes	210	2	
				Microglia	90	5	
				Vascular endothelial cells	180	2	
				Mural cells	60	2	
				Ependymal cells	30	2	
Hippocampal dentate gyrus	Pippeting	SMART-seq	168	CFP^nunc^ qNSC or NPCs	132	6	[73]
				CFP^nuc−^ non-NPCs	26	5	
Striatum	Fluidigm, FACS	SMART-seq2, SMARTer	1208	Neurons (D1-MSN, D2-MSN, interneurons)	368	3	[74]
				Astrocytes	107	1	
				Oligodendrocytes: newly formed (NFO), mature (MO)	43	2	
				Stem cells (NSC, OPC)	20	2	
				Vascular cells (VSMCs, Endothelial)	43	2	
				Immune cells (microglia, macrophage)	119	2	
				Ependymal (ciliated, secretory)	39	2	
Somatosensory cortex, dentate gyrus, hippocampus CA1, corpus callosum, amygdala, hypothalamus, zona incerta, SN-VTA, dorsal horn	Fluidigm, FACS	STRT/C1	5072	Oligodendrocyte precursor cells (OPC)	310	1	[75]
				Committed oligodendrocyte precursors (COP)	140	1	
				Newly formed oligodendrocytes (NFOL1, NFOL2)	219, 293	2	
				Myelin-forming oligodendrocytes (MFOL1, MFOL2)	353, 933	2	
				Mature oligodendrocytes (MOL1, …, MOL6)	126, …, 835	6	
				Vascular and leptomeningeal cells (VLMC)	76	1	

*qNSC* quiescent neural stem cells, *NPC* neural precursor cells, *MSN* medium spiny neurons, *NSC* neural stem cell

Laser capture microdissection is a useful method to isolate cells using a laser pulse [27]. Microscopy is used to verify the position of cells of interest, and then a thermoplastic polymer coating is placed on the tissue over a glass slide. The polymer is melted and then the polymer-cell composition is removed from the tissue. Although specific cells in a tissue are captured, there are some limitations. Contrary to FACS and MACS, LCM is a low-throughput technology. Additionally, LCM relies heavily on cell identification. LCM needs an expert pathologist or cytologist, limiting its extensive application. However, the main advantages of LCM are that it allows researchers to study single-cells within their niche or microenvironment and preserves their spatial location. A cell’s niche is relevant when studying cells with functional diversity linked to spatial location such as brain cells.

## cDNA amplification and sequencing library construction

A single-cell can only supply very limited starting material (about 0.1 pg of mRNA in each cell), so amplification methods are needed to produce high fidelity, high coverage and reliable data [28]. Some of the common reverse transcription and amplification methods used include: SMART-seq/SMART-seq2 (switching mechanism at the 5′ end of the RNA transcript) [9, 29], STRT-seq (single-cell tagged reverse transcription sequencing) [30], CEL-seq (cell expression by linear amplification and sequencing) [31], PMA (Phi29 DNA polymerase-based mRNA transcriptome amplification) [32], SMA (semi-random primed PCR-based mRNA transcriptome amplification procedure) [32], and Quartz-seq [33]. Researchers studying brain scRNA-seq typically use SMART-seq, SMART-seq2, and STRT-seq as outlined in Table 1.

SMART-seq is a reverse transcription and amplification method based on template-switching [9]. First strand cDNAs are created by an oligo(dT)-containing primer, and a few untemplated poly(C) nucleotides are added as overhang at the end of cDNA molecules. The second strand is synthesized by an oligonucleotide primer which can hybridize to the poly(C) overhang, generating full length cDNA products. The purified PCR products can then be used for constructing cDNA libraries. SMART-seq2 is an updated version of SMART-seq [29]. It can significantly improve cDNA yield. In SMART-seq2 protocol, similar to SMART-seq, the first strand is synthesized with 2–5 untemplated nucleotides added at the end of cDNA molecules. Then TSO (template-switching oligonucleotides) with two riboguanosines and a modified guanosine are added to the end of cDNAs. Compared with SMART-seq, SMART-seq2 can produce twofold cDNA products for constructing cDNA libraries.

STRT-seq is also based on templated-switching methods. In this protocol, single-cells are collected and distributed into 96-well PCR plates [30]. Then the cells are lysed by lysis buffer. The first strand is synthesized using oligo(dT) primer and 3–6 cytosines are added to the end of cDNAs. The secondary strand is created using a primer with a cell specific barcode corresponding to each well. After cDNA synthesis, all the products are pooled and then, cDNAs are amplified by a single-primer PCR.

Although reverse transcription and amplification methods can supply sufficient material, they have different levels of amplification bias which are either over-representing or under-representing certain regions of cDNA [28]. For example, SMART-seq, which can provide full-length coverage of cDNAs, has 3′-end bias; but in SMART-seq2, the bias is decreased [9, 34]. STRT-seq has high 5′-end bias [28, 30]. In order to reduce the amplification bias of STRT-seq, UMI (unique molecular identifiers) are integrated in the sequencing primer used for reverse transcription or template switching [35–37]. UMIs are tens of thousands of short, random DNA molecules which are used to label mRNA molecules during reverse transcription prior to amplification. They allow for absolute molecule quantification.

After the cDNA amplification, the cDNA library is constructed. cDNA libraries must be compatible with the sequencing platform. Nextera XT is a widely used library preparation kit. Libraries are generally sequenced by Illumina platforms, such as HiSeq, MiSeq and NextSeq.

## Single-cell RNA-sequencing data analysis

Two important questions which need to be addressed in scRNA-seq assays are the minimum number of cells to be sequenced and the sequencing depth at which the majority of transcripts in a cell can be detected. The answers depend on the experiment’s aims and the nature of the isolated cells. In general, deeper sequencing is required to classify distinct cell types within a homogeneous population of cells [38]. In a sufficiently heterogeneous population, Pollen et al. [39] were able to classify 301 neural cells from the human neural cortex in different developmental stages with as few as 50,000 reads. With numerous experiments with microliter and nanoliter volumes, Wu et al. [40] concluded that beyond one million reads, the number of detected genes per cell varies less than 5%. However, the main variable which will define sequencing depth is the population’s heterogeneity.

### Quality control

As with bulk RNA-seq, the first step in data analysis is quality control. Quality control is generally performed before and after sequencing. Before sequencing, the quality of single-cells is addressed through visual inspection or automated imaging and viability dyes. In contrast with bulk RNA-seq, scRNA-seq protocols result in cells isolated in microwell plates, droplets, or chambers in microfluidic devices. Using microfluidics of droplet technologies, hundreds to thousands of cells can be sequenced in a single run [41, 42]. Due to massive and parallel processing, capture sites may be empty or contain either single or multiple cells. Furthermore, captured cells may be healthy, stressed, broken, or even damaged due to handling. Low quality sites and cells need to be excluded from the experiment since their data may be misleading. Several approaches have been proposed for filtering low quality sites and cells [29, 35, 43–46]. They may be classified into microscopic imaging of individual cells and staining cells with viability dyes.

**Fig. 2:**
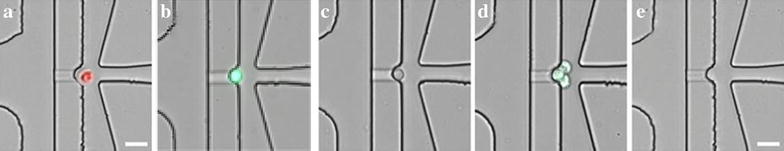
Single-cell widefield representative images acquired by an automated device (C1 Fluidigm chip). **a** Cell stained with ethidium homodimer-1 (EthD-1, *red*) labeling unhealthy or dead cells. **b** Single GFP^+^ cell. **c** Single GFP^−^ cell. **d** Capture site containing three cells. **e** Empty capture site (Figure adapted from [126])

Microscopic cell imaging has proven to identify a high proportion of low quality cells, however this approach is not compatible with all platforms, it is time-consuming, and its automation is challenging. Automated imaging systems rely on visual inspection derived metrics, such as morphology, pixel intensity and frequency. As with other imaging systems, their automation requires a training set of images and machine-learning algorithms, such as Support Vector Machines to discriminate between low and high quality cells. Figure 2 shows representative wide field images captured with an automated imaging device. Staining of dead or viable cells is an effective and relatively fast method, however it can modify a cell’s transcriptional state and alter the experiment’s outcome. After staining cells, an imaging system can determine the cell’s viability by determining pixel intensities as depicted in Fig. 2a.

After sequencing, quality control is performed on raw reads, aligned reads, and across the collection of cells to identify low quality cells. Relevant quality control metrics, similar to those used for bulk RNA-seq, include: per base sequence quality, sequence duplication levels, overrepresented sequences, sequence length distribution, and GC content, among others. Quality control metrics should be calculated for raw reads, as well as for aligned reads. Popular tools for assessing these metrics are FastQC, Kraken [47], and RNA-SeQC [44]. Additionally, parameters such as depth of coverage and library complexity should be addressed. Comparing quality control metrics across all cells is helpful in identifying outliers.

**Fig. 3:**
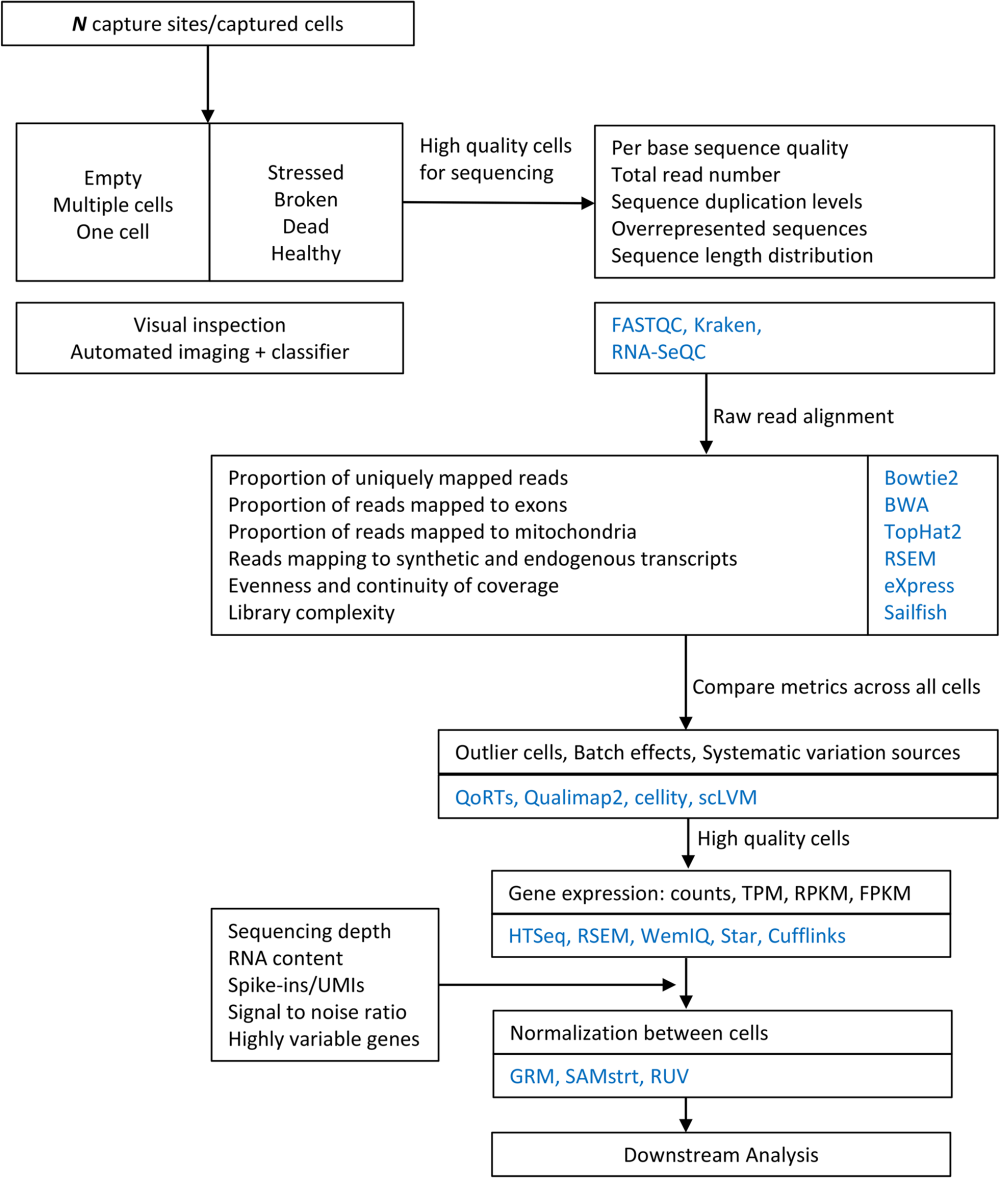
scRNA-seq quality control and expression estimation flow chart

Filtering thresholds are also commonly used for identifying low quality cells after sequencing. Thresholds are typically based on the number of mapped reads and/or on the proportion of detected genes. A comprehensive analysis on low quality cells was published by Ilic et al. [48]. The authors obtained a set of technical and biological measures useful for discriminating low quality cells. Researchers demonstrated that broken cells have a downregulation of genes enriched in gene ontology terms “cytoplasm”, “metabolism”, and “membrane” and an upregulation of genes related to “mitochondrially encoded genes” and “mitochondrially localized proteins”. Due to a compromised cell membrane, broken cells have most likely lost cytoplasmic mRNA while maintaining mRNA enclosed in the mitochondrial membrane, thus resulting in the upregulation of mitochondrially encoded genes. Ilic et al. also proved that empty capture sites and broken cells display lower number of total reads yielding a decreased number of detected genes. Similarly, they concluded that the proportion of duplicated reads is higher in multiple captured cells than in single-cells. Their work was implemented in R and Python libraries available in GitHub repositories. Islam et al. [35] used the total number of detected genes greater than 5000 and at least 85% of cytoplasmic genes (non-mitochondrial and non-ribosomal RNA) as criteria for selecting high quality cells. Figure 3 outlines the various processes involved in scRNA-seq quality control assessment required for discriminating between high and low quality cells. Another useful approach for discriminating low-quality cells is to apply principal component analysis (PCA) to gene expression. The underlying premise of this is that good-quality cells will cluster together and low-quality cells will appear as outliers.

### Gene expression estimation

To quantify gene expression, sequencing reads from high quality cells are aligned to a reference genome and gene counts are computed. If UMIs were used, transcript molecules may be counted directly since the number of UMIs linked to each gene accounts for the number of cDNA molecules associated with it. For non-UMI data, expression may be obtained as counts using tools such as HTSeq [49], RSEM [50], WemIQ [51], and featureCounts [52], among others. Expression is also addressed as relative expression with metrics including transcripts per million mapped reads (TPM), counts per million mapped reads (CPM), reads per kilobase per million mapped reads (RPKM) or fragments per kilobase per million mapped reads (FPKM). Popular tools for assessing relative expression include Cufflinks [53–55], and STAR [56].

**Fig. 4:**
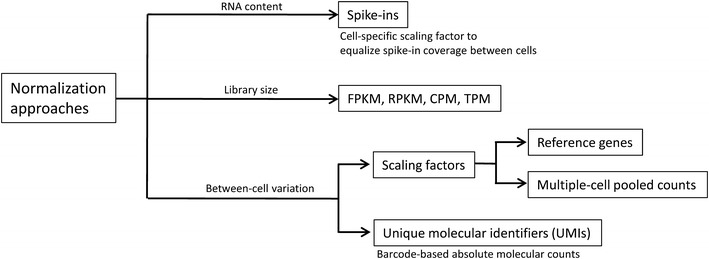
Normalization approaches commonly used in scRNA-seq data analyses

Normalization of scRNA-seq counts is a critical step which allows for expression values to be comparable among cells [57]. Variability between cells may be due to differences in sequencing depth, RNA concentration, GC content, and amplification biases, among others. Normalization methods differ depending on the incorporation of quantitative standards used during library preparation. One approach commonly used in scRNA-seq experiments is adding extrinsic spike-in molecules. Spike-ins are RNA molecules which are either artificially synthesized or obtained from a distant species. Their sequences are known and they are added in a constant concentration to individual cell lysates making them ideal to serve as internal controls. Since the number of spike-in molecules is theoretically the same across all single-cell libraries, they can be used to calculate scaling factors to normalize for differences in RNA concentration between individual cells. The most commonly used artificial spike-in is the External RNA Controls Consortium (ERCC), a set of 96 synthetic RNA molecules based on bacterial sequences [58]. If the ratio between reads mapped to the genome and the number of reads mapped to spike-ins is low, then that cell must be filtered out since this is indicative of low RNA concentration and will bias the results. Normalization approaches are outlined in Fig. 4.

Normalization in the absence of spike-ins or UMIs is generally performed using bulk RNA-seq methods. Several scRNA-seq studies have normalized for sequencing depth by calculating TPM [39, 59] and FPKM/RPKM [60–62]. More sophisticated between-cell normalization approaches include methods where scaling factors are computed, such as in DESeq [63], and edgeR [64]. Median-based normalization methods [43, 65–68] are also widely used. They calculate global scaling factors based on the identification of stable house-keeping genes. Their main premise is that variations in house-keeping gene expression are due to technical sources, however, this is not always valid due to variations in RNA content. The amount of RNA contained in each cell varies intrinsically due to cell-cycle, cell size, and transcriptional gene dynamics [69]. If spike-ins are available, they can be used to estimate individual cell’s RNA content and normalize expression estimates more accurately.

Low amounts of RNA in single-cells are one of the main challenges in scRNA-seq data analysis. There is a negative correlation between the RNA concentration and the number of genes affected by technical noise [43]. Technical noise is generally addressed with the coefficient of variation (CV) in gene expression across control samples, including spike-ins. Technical noise must be accounted for since it may be confounded with biological noise. Determining technical noise is challenging because even housekeeping genes from genetically identical cells may have noisy gene expression [70]. Technical noise may be modeled with a log-normal function to adjust gene expression estimates. Low amounts of RNA present in a single-cell also yield numerous genes with zero or near-zero values. The high frequency of genes with zero counts may affect normalization methods. To overcome this problem, a recent approach, specific for scRNA-seq normalization without spike-ins, proposed a deconvolution method based on pooled counts of genes across multiple cells [71].

In summary, including synthetic spike-ins or unique molecular identifiers with known concentrations (UMIs) has advantages in normalization and expression estimation, however their use still needs to be standardized.

### Downstream analysis

**Fig. 5:**
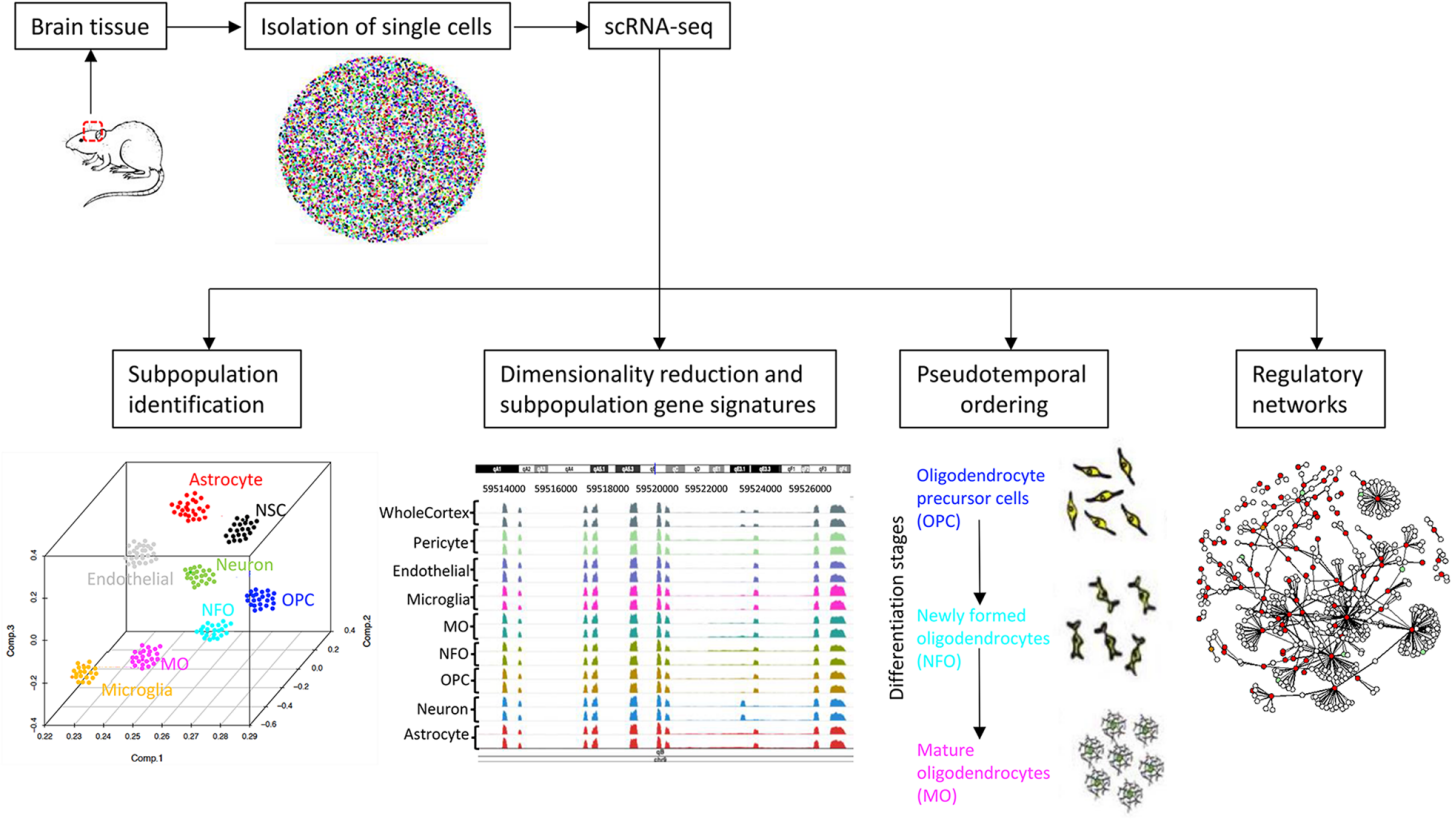
Overview of scRNA-seq downstream analyses

The most common applications for scRNA-seq experiments are: identification of cell types, pseudo-temporal ordering, and network inference. The normalized gene expression count matrix is used for these downstream analyses. A good review on bioinformatics tools useful for single-cell data analysis was published by Poirion et al. [72]. Typical downstream analyses are depicted in Fig. 5. Algorithms used in recent brain scRNA-seq studies are listed in Table 2.

**Table 2:** Data analysis methods used in recent brain scRNA-seq studies

Brain region	Expression measure	Clustering	Refs.
Primary visual cortex (L1, 2/3, 4, 5, 6)	RPKM and counts	PCA, WGCNA, random forests	[21]
Somatosensory cortex and hippocampal CA1	CPM	BackSPIN	[8]
Hippocampal dentate gyrus	TPM	Hierarchical clustering, PCA, Waterfall	[73]
Striatum	CPM	2D-tSNE, rPCA	[74]
Somatosensory cortex, striatum, dentate gyrus, hippocampus CA1, corpus callosum, amygdala, hypothalamus, zona incerta, SN-VTA, dorsal horn	CPM	BackSPINv2	[75]

*RPKM* reads per kilobase of million mapped reads, *CPM* counts per million mapped reads, *TPM* transcripts per million mapped reads, *PCA* principal component analysis, *WGCNA* weighted gene coexpression network analysis, *t-SNE* t-distributed stochastic neighbor embedding, *rPCA* robust principal component analysis

#### Subpopulation identification

Mapping cells individually, rather than in aggregated components as in bulk RNA-seq, makes it feasible to assess the uniqueness of cell subpopulations. Therefore, some of the most popular applications of scRNA-seq is the identification of subpopulations, novel cell subtypes, and rare cell species in a tissue or biological condition [76]. Clustering algorithms are used for grouping cells which have similar gene expression. Cells in each group or cluster are believed to belong to a specific cell subpopulation or cell state. De novo identification of cell-types may be modeled as an unsupervised clustering problem since prior information regarding the number of clusters or marker genes is unknown. Unsupervised clustering methods extensively used to identify cell subpopulations from scRNA-seq samples include PCA and its variants (e.g. Kernel PCA, rPCA) [21, 73, 74], *k*-means, and other distance-based algorithms, such as hierarchical clustering [73]. Common similarity metrics used for distance-based methods are Euclidean distance, Pearson, and Spearman correlation coefficients [39, 77]. A recently developed and frequently used hierarchical clustering method is BackSPIN [8], which allows for biclustering of both genes and cells. The non-linear unsupervised clustering method, t-SNE [78], has also been widely used in scRNA-seq samples [42, 74]. Clustering methods are generally applied to highly variable genes [41, 42], differentially expressed genes (DE) [59, 79], or to highly expressed genes [80]. More sophisticated machine learning methods have been used to overcome the limitations in conventional methods due to the frequency of genes with zero counts. An interesting example is the zero inflated factor analysis (ZIFA), which implements a dimension-reduction approach and uses a latent variable factor model to accommodate zeros [81].

The majority of computational methods for subpopulation identification only address abundant cell types. Therefore, rare cell type identification is a challenging application. Grün et al. [76] developed RaceID, an algorithm for the identification of rare and abundant cell types based on transcript counts obtained with UMIs. RaceID first identifies large clusters defined through k-means clustering of the expression correlation matrix of genes. Next, rare cell types are identified within each cluster by detecting cells whose transcript counts do not display cluster specific expression.

#### Pseudotemporal ordering

scRNA-seq data may be useful for understanding dynamic cellular processes, such as development, reprogramming, differentiation, and disease progression. The underlying premise is that a collection of single-cells will most likely contain cells at different stages during a dynamic process (e.g. differentiation) and profiling their gene expression will allow for the reconstruction of cascades of gene expression changes placing cells in a pseudotemporal order. Pseudotemporal ordering applies machine learning methods to scRNA-seq data to reconstruct cells’ trajectories as they undergo a dynamic biological process. Different algorithms have been implemented for inferring pseudotemporal ordering of single-cells. The first step performed by most temporal ordering algorithms is a dimension reduction such as PCA. For scRNA-seq data, as for bulk RNA-seq, the number of variables or dimensions corresponds to the number of genes. After dimension reduction, if there is prior knowledge of the key maker genes driving the transition between states, methods such as Wanderlust [82] will use graph-based trajectory detection algorithms to order cells along a path. The key marker genes selected for defining a path’s distance may be previously known genes (e.g. genes known to be involved in a differentiation process) or differentially expressed genes. Single-cells may be clustered into subpopulations before temporal ordering.

Several methods which do not require prior knowledge of marker genes have been developed [61, 83, 84]. These methods reconstruct trajectory paths in reduced spaces using several algorithms such as minimum spanning trees (MST), and principal curves. Monocle, developed by Trapnell et al. [61] uses independent component analysis (ICA) for dimension reduction and then constructs an MST to find the paths based on Euclidean distance. Authors achieved a more robust temporal cell ordering when using differentially expressed genes. Monocle2 [85] was recently implemented to overcome the accuracy challenges in trajectory reconstruction. Monocle2 applies reversed graph embedding (RGE) [86] to reconstruct complex single-cell trajectories.

Another popular method for pseudotemporal ordering is Waterfall [73]. Waterfall uses *k*-means and PCA to cluster cells before constructing an MST for ordering cell subpopulations.

#### Finding regulatory networks

Important applications of gene expression profiling have been the identification of co-regulated groups of genes and inferring gene regulatory network dynamics. In co-expression analysis, pairs of genes with similar expression profiles are assumed to be co-regulated and may be part of a signaling cascade. Computational methods have been developed to identify correlated genes or modules [87]. Weighted gene co-expression network analysis (WGCNA) has been a popular network reconstruction tool used for bulk RNA-seq [88]. Xue et al. [89] applied WGCNA to scRNA-seq data obtained from single-cells derived from human and mouse embryos. The authors found functional modules of co-expressed genes for each developmental stage indicating sequential order of transcriptional changes in relevant pathways.

Several mathematical methods such as ordinary differential equations (ODE)-based models and stochastic models have been developed for understanding the dynamics of gene regulation. However, such methods require time-series gene expression profiling, which, for scRNA-seq is unlikely due to sequencing costs. To overcome the lack of temporal data, Ocone et al. [90] proposed a framework which allows the reconstruction of regulatory network dynamics through the combination of dimensionality reduction using diffusion maps [91], pseudo-time single-cell ordering implementing Wanderlust [82], and the generation of ODE-based mathematical transcriptional models. Through their framework, authors were able to reconstruct transcriptional dynamics of specific genes during differentiation of hematopoietic stem cells.

## The application of scRNA-seq in the brain

The mammalian brain is considered to be the most complex organ due to its cellular diversity, the variety and scope of its functions and its transcriptional regulation [92]. Previous studies have aimed at studying the diversity of brain cells through RNA-seq samples from purified populations of cerebral cortex [93, 94]. Recently, scRNA-seq is being used as a tool to assess the brain’s complexity and to identify new cell subpopulations, specific gene signatures, and underlying regulatory networks. This section will provide an overview of relevant scRNA-seq studies related to different types of brain cells. A more detailed description of selected studies is listed in Tables 1 and 2 and depicted in Fig. 1.

### The identification of brain cell types

The brain contains highly complex neural cell types/subtypes. Traditionally, neural cells were identified by morphology, excitability, connectivity and the cell’s location [95]. Recently, scRNA-seq was used to identify different neural types and subtypes, and to discover novel cell-specific markers. For instance, Amit Zeisel et al. [8] sequenced 3005 single-cells and revealed 9 major classes of cells (S1 and CA1 pyramidal neurons, interneurons, oligodendrocytes, astrocytes, microglia, vascular endothelial cells, mural cells and ependymal cells). The authors identified specific novel gene markers for different cell types, for example, S1 pyramidal cells were characterized by *Gm11549* (a long noncoding RNA), hippocampal pyramidal cells by *Spink8* (a serine protease inhibitor), and interneurons by *Pnoc* (prepronociceptin).

Striatum is a subcortical part of the forebrain. The striatal dysfunction can cause many neuropsychiatric disorders, for instance, Parkinson’s and Huntington’s disease, obsessive–compulsive disorder, and autism [96, 97]. Traditionally, the neuronal composition of the striatum has been defined by mostly medium spiny neurons (MSN) and a small population of interneurons [74]. MSNs have been classified anatomically and functionally into D1 and D2 MSNs [98] however, striatal diversity has not been assessed.

Ozgun Gokce et al. [74] used two approaches: microfluidic single-cell RNA sequencing (MIC-scRNA-seq) and single-cell isolation by fluorescence-activated cell sorting (FACS-scRNA-seq) to analyze the transcriptomes of 1028 single striatal cells. The transcriptomes revealed ten different cell subpopulations including neurons, astrocytes, oligodendrocytes, stem cells, immune, ependymal, and vascular cells. Through robust PCA, novel gene markers were found to discriminate between D1 and D2 MSN cells.

Neural stem cells (NSCs) can self-renew and produce neural cell types, including neurons, astrocytes and oligodendrocytes [99, 100]. NSCs maintain a balance between quiescent and activated states [101, 102]. If the brain is injured, endogenous NSC will be activated to repair brain tissue [103]. Previous works were limited by small number of factors analyzed and mixed cell populations. It was not completely understood how the NSCs became activated. Recently, two studies have used single-cell methods to examine the activation of dormant neuron stem cells after injury. In one study, Llorens-Bobadilla and colleagues investigated the characteristics of the activation of dormant NSCs after brain injury [22]. The authors identified NSCs in quiescent and active states and uncovered the progression of activation using single-cell sequencing. They identified new gene markers of NSCs subpopulations and they found that, during brain ischemia, dormant NSCs proceed to activation via interferon gamma signaling. Another study also showed that central nervous system (CNS) injury could activate CD133^+^ quiescent NSCs. Luo et al. [104] demonstrated that vascular endothelial growth factor (VEGF) could activate CD133^+^ ependymal neural stem cell (NSCs), and together with basic fibroblast growth factor, elicit neural lineage differentiation and migration. In a recent study, Dulken et al. [57] sequenced 329 high quality single-cells sorted by FACS from four different populations [astrocytes, quiescent neural stem cells (qNSC), activated neural stem cells (aNSCs), and neural precursor cells (NPCs)] within the sub-ventricular zone of adult mice. Through PCA, authors were able to discriminate quiescent cell types (astrocytes and qNSCs) from active and proliferative cell types (aNSCs and NPCs). Interestingly, authors compared their single-cell transcriptomes with those from similar cells [NSCs and transit amplifying progenitors (TAPs)] sorted with different cell markers [22]. To be able to compare single-cell datasets processed in different batches and thus with dissimilar library preparations and sequencing depths, Dulken et al. mapped Llorens-Bobadilla and colleagues’ datasets using their own pipeline and then performed PCA with the most variable genes. Additionally, they performed pseudo-time ordering using Monocle with their consensus-ordering genes and found similar dynamic gene expression related to quiescence and activation of NSCs. Through this meta-analysis, authors were able to observe a high correlation between NSCs from both studies in spite of divergent isolation methods and batch effects.

Oligodendrocytes were considered an important functionally homogeneous population in the CNS, however these cell’s morphologies are diverse [105]. It is unclear whether the diversity in morphology is due to oligodendrocytes interacting with the local environment during maturation or due to their intrinsic functional heterogeneity [106, 107]. Marques et al. [75] isolated single-cells from 10 different regions of juvenile and adult mice CNS by FACS and sequenced 5072 oligodendrocytes by scRNA-seq. The authors identified 13 distinct subpopulations from which 12 represent differentiation stages from oligodendrocyte precursor cells to mature oligodendrocytes. The fine differentiation stages were identified using t-SNE for dimensionality reduction and the biclustering tool BackSPIN2 for pseudo-time analysis. Thereby, using scRNA-seq methods, the authors revealed the dynamics of the differentiation and maturation of oligodendrocytes.

It is difficult to interrogate the underlying transcription landscape of individual neurons. Previously, many studies of single adult human neurons were dependent on the availability of freshly isolated neurosurgical tissues from limited regional samples [109]. Although freshly isolated neurosurgical tissues are better for analyzing single neurons, postmortem tissues can provide more input sample. Lake and colleagues developed a new method which can sequence and quantify RNA in isolated neuron nuclei from postmortem brains [108]. They dissected six distinct regions of the cerebral cortex, and produced 3227 sets of single-neuron RNA-seq data. After clustering and classification, 16 neuronal subtypes were identified and were evaluated by known markers and cortical cytoarchitecture.

### The regulation of brain developments by long non-coding RNAs (lncRNAs)

Studies have revealed thousands of lncRNAs in mammalian transcriptomes [110]. lncRNAs are not well conserved during evolution [111], but the promoters of lncRNAs are more conserved than protein coding genes [112, 113]. lncRNAs have tissue specific expression in human brain [114, 115] and have been shown to be involved in the regulation of brain diseases and neurodevelopmental disorders [116, 117]. Previous studies based on bulk tissues suggested that the expression levels of lncRNAs are lower than those of protein coding genes [114, 118]; however, it is unknown whether lncRNAs are expressed at low levels in all cells [119].

Researchers have studied the expression of lncRNAs in purified mouse brain cells and found their role in fate determination of oligodendrocyte precursor cells (OPC) [120]. Recent approaches are now aiming at addressing lncRNAs in brain scRNA-seq samples. Liu et al. [119] used scRNA-seq to analyze lncRNAs in the developing human neocortex. The authors isolated total RNA from 276 single-cells of different stages of human neocortex development and analyzed their transcriptomes. To evaluate if lncRNAs were expressed at high levels in subpopulations of cells, the authors used the lncRNA:mRNA median ratio which compares the median expression of lncRNAs to the median expression of mRNA. Compared with lncRNAs from bulk tissue (the median lncRNA:mRNA ratio was 0.31), many lncRNAs were abundantly expressed in individual cells (in single-cells, 32.2% of cells’ median lncRNA:mRNA ratio exceeded 1.0). The authors found that lncRNA *LOC646329* was enriched in the ventricular zone, where most radial glia reside. When *LOC646329* was knocked down, the propagation of U87 cells was reduced. Results suggest that lncRNAs might regulate cell proliferation.

## Future perspectives

In summary, scRNA-seq is a powerful tool that will allow researchers to address human brain complexity by identifying cell subpopulations and elucidating specific functions. scRNA-seq has a higher resolution than bulk RNA-seq and allows us to better understand cellular heterogeneity and how it changes during dynamic processes, such as development, differentiation and disease progression. Major resolution, however, makes samples more vulnerable to disturbances and confounding effects. Experimental and computational methods are being developed to overcome challenges posed by detecting single-cell signal in the presence of intrinsic noise and technical variability. Recently, chromatin accessibility [121, 122], chromatin conformation [123], and DNA methylation [124] with single-cell resolution were successfully implemented. Single-cell DNA/RNA-seq approaches will allow scientists to simultaneously assess the genomic, epigenomic, and transcriptomic states of individual cells in biological processes. Single-cell sequencing will be expanded to also address metabolomics in order to construct a more complete picture of a cell.

## Abbreviations

CEL-seq: cell expression by linear amplification and sequencing
CNS: central nervous system
COP: committed oligodendrocyte precursors
CPM: counts per million mapped reads
CV: coefficient of variation
ERCC: External RNA Controls Consortium
FACS: fluorescence-activated cell sorting
FPKM: fragments per kilobase per million mapped reads
GFP: green fluorescent protein
GRM: gamma regression model
ICA: independent component analysis
LCM: laser capture microdissection
lncRNA: long non-coding RNA
MACS: magnetic-activated cell sorting
MO: mature oligodendrocytes
MSN: medium spiny neurons
MST: minimum spanning tree
NFO: newly formed oligodendrocytes
NPC: neural precursor cells
NSC: neural stem cells
OPC: oligodendrocyte precursor cells
PCA: principal component analysis
PMA: Phi29 DNA polymerase-based mRNA transcriptome amplification
qNSC: quiescent neural stem cells
rPCA: robust principal component analysis
RPKM: reads per kilobase per million mapped reads
RUV: removed unwanted variation
scRNA-seq: single-cell RNA-sequencing
SMA: semi-random primed PCR-based mRNA transcriptome amplification procedure
SMART-seq: switching mechanism at the 5′ end of the RNA transcript sequencing
STRT-seq: single-cell tagged reverse transcription sequencing
TPM: transcripts per million mapped reads
TSO: template-switching oligonucleotides
UMI: unique molecular identifiers
VEGF: vascular endothelial growth factor
VLMC: vascular and leptomeningeal cells
VSMCs: vascular smooth muscle cells
WGCNA: weighted gene co-expression network analysis
ZIFA: zero inflated factor analysis
